# HbA1c-Based Metabolic Stratification: A Retrospective Study of Clinical and Biological Differences Across Normoglycemic, Prediabetic, and Diabetic Subjects

**DOI:** 10.3390/diagnostics16040566

**Published:** 2026-02-13

**Authors:** Mihaela Simona Popoviciu, Timea Claudia Ghitea, Carmen Pantis, Nicolae Ovidiu Pop, Roxana Daniela Brata

**Affiliations:** 1 Department of Preclinical Disciplines, Faculty of Medicine and Pharmacy, University of Oradea, 1 Decembrie, 410028 Oradea, Romania; mihaela.popoviciu@didactic.uoradea.ro; 2 Department of Internal Medicine II, Diabetes Mellitus, Clinical County Emergency Hospital of Oradea, 410167 Oradea, Romania; 3 Pharmacy Department, Faculty of Medicine and Pharmacy, University of Oradea, 1 Decembrie, 410028 Oradea, Romania; 4 Department of Medical Disciplines, Faculty of Medicine and Pharmacy, University of Oradea, 1 Decembrie, 410028 Oradea, Romania; pop.nicolaeovidiu@didactic.uoradea.ro (N.O.P.); brata.roxanadaniela@didactic.uoradea.ro (R.D.B.)

**Keywords:** HbA1c, prediabetes, diabetes mellitus, metabolic stratification, microvascular complications, risk estimation, peripheral neuropathy, retinopathy, cardiometabolic risk

## Abstract

**Background:** Glycated hemoglobin (HbA1c) is widely used for the diagnosis and monitoring of diabetes mellitus. However, its potential role as a marker of metabolic stratification and early complication risk beyond diagnostic thresholds remains insufficiently explored. Prediabetes is often considered a transitional state, although growing evidence suggests the presence of early metabolic and microvascular alterations. **Methods:** In this cross-sectional observational study, adult subjects were stratified into three groups based on HbA1c levels: normoglycemic (HbA1c < 5.7%), prediabetic (HbA1c 5.7–6.4%), and diabetic (HbA1c ≥ 6.5%). Demographic data, metabolic parameters, cardiometabolic comorbidities, and diabetes-related complications were analyzed. Group differences were assessed using appropriate statistical tests. Cumulative complication burden was evaluated, and risk estimation was performed using univariate logistic regression models to calculate odds ratios (ORs) and 95% confidence intervals (CIs). **Results:** A total of 839 subjects were included in the analysis. HbA1c and fasting glucose levels increased progressively across groups (*p* < 0.001). Prediabetic individuals already exhibited a higher prevalence of peripheral neuropathy and retinopathy compared to normoglycemic subjects, while diabetic patients showed the highest overall complication burden. The cumulative number of complications increased significantly across HbA1c-based groups (*p* = 0.033). Risk estimation analyses revealed increased odds for presenting at least one complication and for peripheral neuropathy in diabetic subjects, with intermediate risk levels observed in the prediabetic group. **Conclusions:** HbA1c-based stratification captures meaningful differences in metabolic status, complication burden, and estimated risk across normoglycemic, prediabetic, and diabetic individuals. Prediabetes is associated with detectable microvascular involvement and higher complication risk. Given the cross-sectional design, these findings reflect associations rather than causal relationships. These findings highlight the potential clinical value of HbA1c for early risk assessment and targeted preventive strategies.

## 1. Introduction

Diabetes mellitus represents a major global public health challenge, with a continuously increasing prevalence and a substantial burden of chronic complications [1]. Glycated hemoglobin (HbA1c) is widely used as a diagnostic and monitoring tool, reflecting long-term glycemic exposure and serving as a cornerstone in diabetes management [2,3]. Beyond its diagnostic utility, HbA1c has been increasingly recognized as a marker of metabolic stress associated with cardiovascular and microvascular risk [4].

Prediabetes, defined by intermediate HbA1c levels, affects a significant proportion of the adult population and is traditionally viewed as a transitional state preceding overt diabetes. However, growing evidence suggests that prediabetes may already be associated with adverse metabolic alterations and early tissue damage. Subclinical inflammation, endothelial dysfunction, and glycemic variability have been implicated as potential mechanisms contributing to early microvascular injury even before diagnostic thresholds for diabetes are reached [5,6,7].

Despite this emerging evidence, clinical practice continues to rely heavily on dichotomous diagnostic criteria, potentially underestimating the biological and clinical relevance of intermediate glycemic states. The extent to which HbA1c-based stratification captures differences in complication burden and risk across normoglycemic, prediabetic, and diabetic individuals remains incompletely characterized, particularly in real-world clinical cohorts [8,9,10].

Microvascular complications such as peripheral neuropathy, retinopathy, and nephropathy are traditionally associated with established diabetes; however, their presence in individuals with prediabetes has been increasingly reported. In addition, the cumulative burden of multiple complications may offer a more comprehensive measure of metabolic damage than isolated outcomes. Risk estimation approaches, including odds ratio–based analyses, may further enhance the clinical applicability of HbA1c stratification by providing quantitative assessments of complication susceptibility [11,12,13].

Therefore, the aim of the present study was to evaluate the clinical relevance of HbA1c-based stratification by comparing metabolic characteristics, the prevalence and cumulative burden of diabetes-related complications, and estimated complication risk across normoglycemic, prediabetic, and diabetic subjects. By adopting a combined descriptive and risk-based analytical framework, this study seeks to contribute to a more nuanced understanding of glycemic status as a continuous spectrum of metabolic risk. Although the association between hyperglycemia and complications is well established, fewer studies have examined HbA1c-based stratification as a continuum framework. Such an approach may capture cumulative complication burden and early microvascular involvement in real-world populations. Unlike many previous studies focusing on single outcomes, our approach integrates cumulative complication indices and risk estimation within a real-world clinical cohort, providing a broader perspective on HbA1c-based metabolic stratification.

## 2. Materials and Methods

### 2.1. Study Design and Population

This cross-sectional observational study included adult subjects evaluated for glycemic status and diabetes-related complications. Clinical and laboratory data were retrospectively collected from an institutional database. Subjects with complete information on HbA1c levels and key clinical variables were eligible for inclusion in the analysis.

Clinical and laboratory data were retrospectively collected from the electronic medical database of the Clinical County Emergency Hospital of Oradea, Romania, a tertiary-care center serving a large regional population. Data were collected for the period January 2023–December 2025.

Participants were stratified into three groups according to HbA1c levels, based on established diagnostic criteria:

- Normoglycemic group: HbA1c < 5.7%
- Prediabetic group: HbA1c 5.7–6.4%
- Diabetic group: HbA1c ≥ 6.5%

The database did not consistently distinguish between type 1 and type 2 diabetes for all participants. Given the age distribution and real-world clinical setting, most diabetic subjects were presumed to have type 2 diabetes. However, formal classification was not used as a stratification variable.

Due to the retrospective nature of the study, the exact temporal alignment between HbA1c measurement and clinical diagnosis of complications could not be standardized for all participants. In most cases, HbA1c values reflected routine evaluations performed in close temporal proximity to clinical assessment.

### 2.2. Clinical and Metabolic Variables

Demographic data included age and sex. Metabolic assessment comprised HbA1c levels and fasting plasma glucose. The presence of major cardiometabolic comorbidities, including hypertension, dyslipidemia, and obesity, was recorded based on clinical diagnosis and/or documented medical history.

Obesity was categorized according to standard body mass index (BMI) classifications into normal weight, overweight, and obesity grades I–III.

### 2.3. Assessment of Diabetes-Related Complications

Diabetes-related complications were evaluated based on documented clinical diagnoses and included:

- Peripheral neuropathy,
- Diabetic retinopathy,
- Diabetic nephropathy,
- Peripheral arteriopathy.

In addition to individual complications, a cumulative complication score was calculated for each subject, representing the total number of documented diabetes-related complications.

Peripheral arteriopathy was included despite its lower prevalence because it represents a clinically relevant vascular complication and contributes to the overall cumulative burden of diabetes-related damage.

For risk estimation analyses, a binary outcome variable indicating the presence of at least one complication was defined.

### 2.4. Statistical Analysis

Continuous variables were tested for normality and are presented as mean ± standard deviation or median, as appropriate. Differences between HbA1c-based groups were assessed using one-way analysis of variance (ANOVA) for normally distributed variables or the Kruskal–Wallis test for non-normally distributed variables.

Categorical variables are presented as numbers and percentages, and group comparisons were performed using the chi-square test.

Risk estimation was conducted using univariate logistic regression models, with normoglycemic subjects serving as the reference group. Odds ratios (ORs) and corresponding 95% confidence intervals (CIs) were calculated for the presence of at least one complication, peripheral neuropathy, and retinopathy. Because risk estimation relied on univariate models, potential confounding by variables such as age or hypertension cannot be excluded.

All statistical analyses were performed using IBM SPSS Statistics (version 30, IBM Corp., Armonk, NY, USA). A two-sided *p*-value < 0.05 was considered statistically significant.

### 2.5. Ethical Considerations

The study was conducted in accordance with the Declaration of Helsinki and approved by the Institutional Ethics Committee of the University of Oradea (protocol CEFMF/1, 31 January 2023).

## 3. Results

### 3.1. General Characteristics of the Cohort

The study population was stratified into three groups according to HbA1c levels: normoglycemic subjects (HbA1c < 5.7%), prediabetic subjects (HbA1c 5.7–6.4%), and diabetic subjects (HbA1c ≥ 6.5%).

A total of 839 participants were included in the analysis, of whom 86 (10.2%) were normoglycemic, 195 (23.2%) were classified as prediabetic, and 558 (66.5%) as diabetic.

Table 1 presents the demographic and clinical characteristics of the study population stratified according to HbA1c levels.

Mean age differed significantly across the three groups, with normoglycemic participants being slightly younger compared to prediabetic and diabetic subjects (*p* = 0.015). Sex distribution was comparable among groups, with no significant differences observed (*p* = 0.885).

As expected, HbA1c levels increased progressively across groups, confirming the validity of the stratification (*p* = 0.001). Fasting glucose levels also showed a significant stepwise increase from normoglycemic to diabetic subjects (*p* = 0.001), reflecting worsening glycemic control.

The prevalence of hypertension increased significantly with higher HbA1c categories (*p* = 0.001), highlighting the association between glycemic status and cardiovascular risk. In contrast, no significant differences were observed among groups with respect to obesity categories (*p* = 0.689) or dyslipidemia status (*p* = 0.674). The absence of significant differences in obesity and dyslipidemia suggests that HbA1c-based stratification captures risk beyond traditional metabolic comorbidities.

### 3.2. Differences in Metabolic and Diabetes-Related Complications Across HbA1c-Based Groups

Differences in diabetes-related complications across HbA1c-defined groups are summarized in this section. A progressive increase in the prevalence of several chronic complications was observed from normoglycemic to diabetic subjects (Table 2).

Peripheral neuropathy was already present in the prediabetic group, with a higher prevalence compared to normoglycemic subjects, and increased further in the diabetic group (*p* = 0.043).

Similarly, retinopathy showed a significant increase across HbA1c categories, with a marked rise in diabetic subjects compared to both normoglycemic and prediabetic individuals (*p* = 0.032).

In contrast, the prevalence of nephropathy did not differ significantly between groups (*p* = 0.543), suggesting a less pronounced association with early glycemic deterioration in this cohort.

Peripheral arteriopathy was infrequent in normoglycemic and prediabetic subjects and became more prevalent only in the diabetic group; however, this difference did not reach statistical significance (*p* = 0.488).

**Figure 1:**
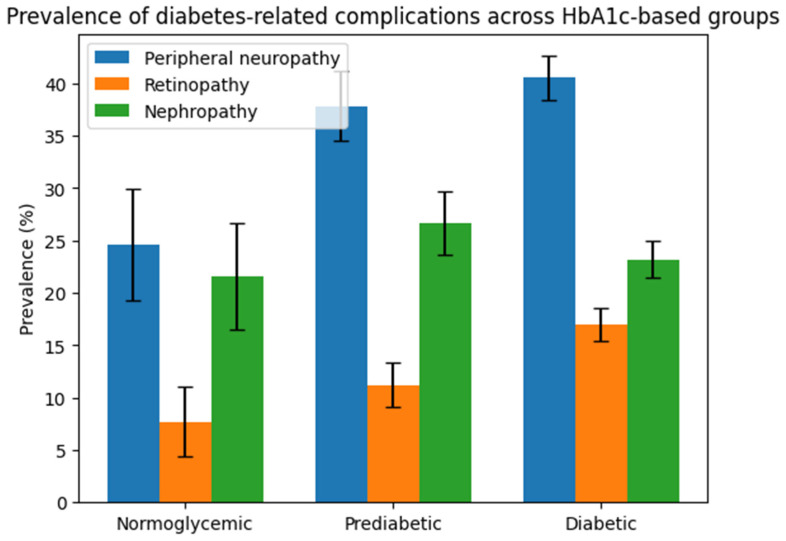
Prevalence of diabetes-related complications across HbA1c-based groups. Bar chart illustrating the prevalence of peripheral neuropathy, retinopathy, and nephropathy across HbA1c-based groups (normoglycemic, prediabetic, and diabetic subjects). Data are expressed as percentages, and error bars represent the standard deviation of the proportion. Group differences were evaluated using the chi-square test; statistically significant differences between groups were considered at *p* < 0.05.

The prevalence of diabetes-related complications across HbA1c-based groups is presented in Figure 1.

### 3.3. Cumulative Burden of Diabetes-Related Complications Across HbA1c-Based Groups

The cumulative number of diabetes-related complications differed significantly across HbA1c-based groups, as presented in Table 3.

Normoglycemic subjects presented the lowest burden of complications, with a mean number of 0.55 ± 0.61 complications per subject. This value increased in the prediabetic group (0.78 ± 0.80) and was highest among diabetic subjects (0.84 ± 0.81).

**Figure 2:**
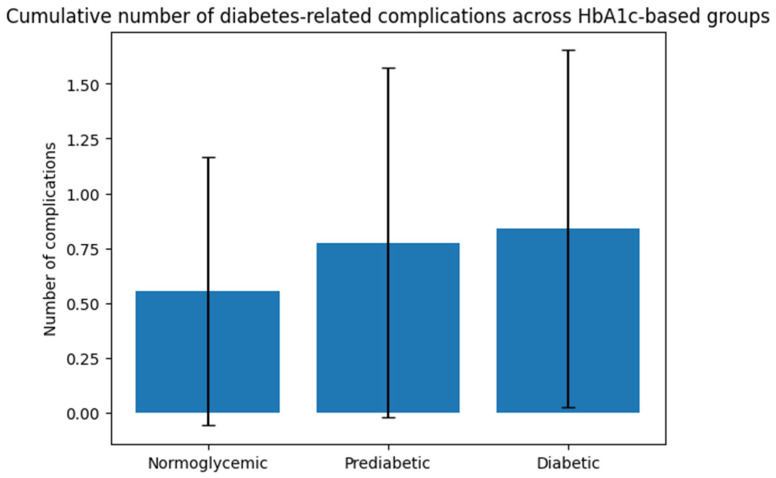
Cumulative number of diabetes-related complications across HbA1c-based groups. Bar chart illustrating the mean cumulative number of diabetes-related complications across HbA1c-based groups (normoglycemic, prediabetic, and diabetic subjects). Error bars represent standard deviation. Group differences were evaluated using the Kruskal–Wallis test due to non-normal distribution of the data. A *p* value < 0.05 was considered statistically significant.

A statistically significant difference in the cumulative number of complications was observed across the three groups (*p* = 0.033), indicating a progressive increase in overall complication burden with worsening glycemic status (Figure 2).

### 3.4. Estimation of Complication Risk Across HbA1c-Based Groups

To further evaluate the association between glycemic status and complication burden, risk estimation analyses were performed using HbA1c-based groups as predictors.

Compared to normoglycemic subjects, individuals in the prediabetic group exhibited an increased risk of presenting at least one diabetes-related complication. This risk was further accentuated in the diabetic group, indicating a stepwise increase in complication susceptibility with worsening glycemic control.

The estimated risk of peripheral neuropathy was significantly higher in prediabetic subjects compared to normoglycemic individuals and increased further in diabetic patients. A similar pattern was observed for retinopathy, supporting a progressive microvascular risk gradient across HbA1c-defined categories.

These findings suggest that HbA1c-based stratification allows not only descriptive characterization of metabolic status but also meaningful estimation of complication risk, even at glycemic levels below the diagnostic threshold for diabetes (Table 4).

**Figure 3:**
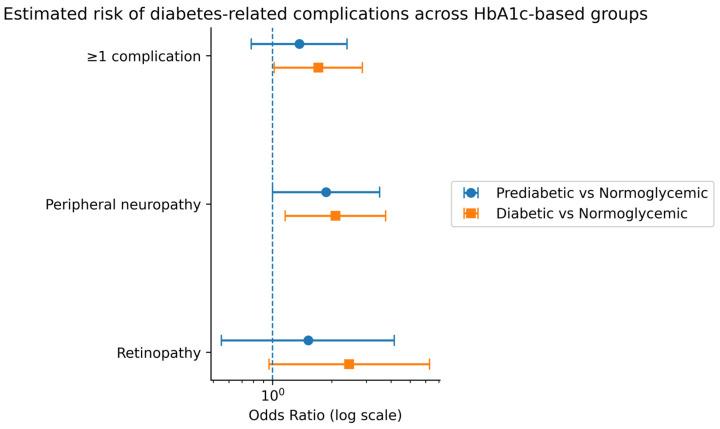
Forest plot of estimated risk of diabetes-related complications across HbA1c-based groups. The plot shows odds ratios (ORs) with 95% confidence intervals (CIs) derived from univariate logistic regression models. Normoglycemic subjects (HbA1c < 5.7%) served as the reference group. An OR > 1 indicates higher odds of complications compared to the reference group. Associations were considered statistically significant when the 95% CI did not include 1.0 (*p* < 0.05).

The estimated risk of diabetes-related complications across HbA1c-based groups is presented in Figure 3.

## 4. Discussion

In this study, we evaluated the clinical relevance of HbA1c-based stratification by analyzing metabolic characteristics, the prevalence of diabetes-related complications, cumulative complication burden, and estimated complication risk across normoglycemic, prediabetic, and diabetic subjects. The retrospective design limited precise temporal mapping between HbA1c levels and complication onset. Therefore, HbA1c should be interpreted as a marker of metabolic status rather than a direct temporal predictor of complications. Our findings highlight a progressive metabolic and microvascular risk continuum, extending well below the diagnostic threshold for diabetes.

### 4.1. HbA1c as a Marker of Metabolic Stratification Beyond Diagnosis

As expected, HbA1c and fasting glucose levels increased stepwise across the three groups, validating the applied stratification. Importantly, this glycemic gradient was accompanied by significant differences in age and hypertension prevalence, supporting the concept that HbA1c reflects broader cardiometabolic risk rather than isolated glycemic control [14,15,16].

Notably, sex distribution, obesity categories, and dyslipidemia prevalence did not differ significantly between groups, suggesting that the observed differences in complication burden are not primarily driven by these factors. This strengthens the role of HbA1c as an independent marker of metabolic vulnerability in this cohort [17,18,19,20].

### 4.2. Early Microvascular Involvement in Prediabetes

One of the key findings of this study is the presence of diabetes-related complications already in the prediabetic group. Both peripheral neuropathy and retinopathy showed a significantly higher prevalence in prediabetic subjects compared to normoglycemic individuals, indicating that microvascular damage may begin early in the course of glycemic deterioration [21,22,23,24]. The absence of significant differences may reflect lower sensitivity of clinical detection at early stages or limited statistical power for less frequent outcomes.

These observations are consistent with previous reports suggesting that chronic low-grade hyperglycemia, glycemic variability, and associated metabolic disturbances can contribute to endothelial dysfunction and neural injury even before overt diabetes is diagnosed. Our results support the notion that prediabetes represents an active pathological state rather than a benign transitional condition [25,26,27,28].

### 4.3. Progressive Accumulation of Complication Burden

Beyond individual complications, we observed a significant increase in the cumulative number of diabetes-related complications across HbA1c-based groups. Prediabetic subjects already exhibited a higher mean number of complications compared to normoglycemic individuals, while diabetic patients showed the highest overall burden [29,30].

This gradual accumulation suggests that metabolic deterioration reflects quantitative rather than qualitative changes. Diabetes may represent the upper end of a continuous risk spectrum. The use of cumulative complication indices may therefore provide additional clinical insight beyond binary outcome assessment [31,32].

### 4.4. Risk Estimation and Clinical Implications

Risk estimation analyses further supported a stepwise increase in complication susceptibility across HbA1c categories. Prediabetic subjects demonstrated increased odds for peripheral neuropathy and for presenting at least one complication, while diabetic subjects showed significantly higher risks across multiple outcomes [33,34,35].

The forest plot analysis visually reinforces this gradient of risk and underscores the potential utility of HbA1c-based grouping for risk stratification in clinical practice. Even unadjusted risk estimates revealed clinically meaningful associations, highlighting the relevance of early identification and monitoring of individuals with intermediate HbA1c levels.

### 4.5. Clinical Relevance and Potential Implications

Taken together, these findings suggest that relying solely on diagnostic thresholds may underestimate the true burden of metabolic and microvascular damage in prediabetes. HbA1c-based stratification may therefore serve as a valuable tool not only for diagnosis but also for early risk assessment and targeted preventive strategies [36].

Early lifestyle or pharmacological interventions in prediabetic individuals could potentially mitigate the progression of both glycemic deterioration and complication development, an aspect that warrants further prospective investigation.

The novelty of our study lies less in identifying new complications and more in demonstrating how HbA1c-based stratification captures a graded continuum of metabolic vulnerability in routine clinical practice. By integrating cumulative complication burden and risk estimation, our findings support a shift from binary diagnostic thinking toward spectrum-based metabolic risk assessment.

### 4.6. Strengths and Limitations

The strengths of this study include the relatively large sample size, the comprehensive assessment of multiple diabetes-related complications, and the combined use of descriptive, cumulative, and risk-based analytical approaches. However, several limitations should be acknowledged. The cross-sectional design precludes causal inference, and the lack of adjustment for disease duration, treatment effects, or additional metabolic markers may have influenced risk estimates. Future longitudinal studies are needed to confirm the temporal relationship between HbA1c progression and complication development.

The lack of precise temporal standardization between HbA1c measurement and complication assessment may have introduced misclassification or temporal bias.

The absence of systematic classification into type 1 and type 2 diabetes represents a limitation, as pathophysiology and complication trajectories may differ between these entities. This may limit pathophysiological interpretation of complication patterns.

Another important limitation is the absence of detailed data on antidiabetic treatment, insulin use, and other metabolic therapies. Pharmacological interventions can substantially influence HbA1c levels and complication risk. Consequently, observed associations may partly reflect treatment effects rather than intrinsic glycemic status alone. Similarly, incomplete characterization of comorbidity burden beyond major cardiometabolic conditions may have introduced residual confounding. These factors should be carefully considered when interpreting our findings.

Our findings demonstrate that HbA1c-based stratification captures meaningful differences in metabolic status, complication burden, and estimated risk across normoglycemic, prediabetic, and diabetic individuals. Prediabetes is associated with detectable microvascular involvement and increased complication risk, supporting the concept of a continuous metabolic risk spectrum rather than discrete diagnostic categories.

### 4.7. Future Directions

Future studies should include prospective designs, multivariable modeling, and additional metabolic or inflammatory biomarkers to refine risk prediction.

## 5. Conclusions

This study demonstrates that HbA1c-based stratification is associated with meaningful differences in metabolic status, diabetes-related complications, cumulative complication burden, and estimated complication risk. Importantly, individuals classified as prediabetic already exhibit signs of microvascular involvement and an increased susceptibility to complications compared to normoglycemic subjects.

The progressive increase in complication prevalence and risk across HbA1c categories supports the concept of a continuous metabolic risk spectrum rather than a strict threshold-based separation. These findings highlight the potential clinical value of HbA1c not only as a diagnostic marker but also as a tool for early risk assessment and stratification.

Early identification and targeted intervention in individuals with intermediate HbA1c levels may help reduce the burden of diabetes-related complications. From a translational perspective, HbA1c-based stratification may help clinicians identify individuals at elevated metabolic risk even before overt diabetes develops. Further longitudinal studies are warranted to confirm these associations and to evaluate the impact of early preventive strategies on long-term clinical outcomes. These associations should be interpreted in the context of the study’s cross-sectional design.

## Figures and Tables

**Table 1:** Demographic and clinical characteristics of the study population stratified by HbA1c categories.

Parameters		Groups			*p*
		HbA1c < 5.7%	HbA1c 5.7–6.4%	HbA1c ≥ 6.5%	
		Count (%)	Count (%)	Count (%)	
Age (years)		60.1 ± 11.5	63.5 ± 10.2	63.4 ± 10.0	0.015
Sex	Male	31 (47.7%)	101 (47.2%)	275 (49.1%)	0.885
	Female	34 (52.3%)	113 (52.8%)	285 (50.9%)	
HbA1c (%)		5.50 ± 0.20	6.10 ± 0.18	8.18 ± 1.74	0.001
Fasting glucose (mg/dL)		106.7 ± 20.8	123.1 ± 19.4	176.0 ± 62.2	0.001
HTN	No	23 (35.4%)	73 (34.1%)	186 (33.2%)	0.001
	Yes	42 (64.6%)	141 (65.9%)	374 (66.8%)	
Obesity	Normal	17 (26.2%)	48 (22.4%)	127 (22.7%)	0.689
	Overweight	17 (26.2%)	55 (25.7%)	126 (22.5%)	
	Obesity degree I	20 (30.8%)	61 (28.5%)	189 (33.8%)	
	Obesity degree II	6 (9.2%)	38 (17.8%)	80 (14.3%)	
	Obesity degree III	5 (7.7%)	12 (5.6%)	38 (6.8%)	
Dyslipidemia	No	27 (41.5%)	76 (35.5%)	180 (32.1%)	0.674
	Yes	38 (58.5%)	138 (64.5%)	380 (67.9%)	

Data are presented as mean ± standard deviation for continuous variables and number (percentage) for categorical variables. *p*-values for continuous variables were calculated using one-way ANOVA. *p*-values for categorical variables were calculated using the chi-square test.

**Table 2:** Prevalence of diabetes-related complications across HbA1c-based groups.

Complication	Normoglycemic (HbA1c < 5.7%) <br> *n* = 65	Prediabetic (HbA1c 5.7–6.4%) <br> *n* = 214	Diabetic (HbA1c ≥ 6.5%) <br> *n* = 560	*p*-Value
Peripheral neuropathy, *n* (%)	16 (24.6%)	81 (37.9%)	227 (40.5%)	0.043
Retinopathy, *n* (%)	5 (7.7%)	24 (11.2%)	95 (17.0%)	0.032
Nephropathy, *n* (%)	14 (21.5%)	57 (26.6%)	130 (23.2%)	0.543
Peripheral arteriopathy, *n* (%)	1 (1.5%)	4 (1.9%)	18 (3.2%)	0.488

Data are presented as number (percentage). *p*-values were calculated using the chi-square test.

**Table 3:** Cumulative number of diabetes-related complications across HbA1c-based groups.

Group	*n*	Mean Number of Complications	SD	Median	*p*-Value
Normoglycemic (HbA1c < 5.7%)	65	0.55	0.61	0	0.033 *
Prediabetic (HbA1c 5.7–6.4%)	214	0.78	0.80	1	
Diabetic (HbA1c ≥ 6.5%)	560	0.84	0.81	1	

* *p*-value calculated using the Kruskal–Wallis test. Data are presented as mean ± standard deviation and median, as appropriate. Differences between groups were assessed using the Kruskal–Wallis test due to non-normal distribution of the number of complications.

**Table 4:** Estimated risk of diabetes-related complications across HbA1c-based groups.

Outcome	Prediabetic vs. Normoglycemic OR (95% CI)	Diabetic vs. Normoglycemic OR (95% CI)
≥1 complication	1.37 (0.78–2.39)	1.71 (1.02–2.86)
Peripheral neuropathy	1.87 (1.00–3.50)	2.09 (1.16–3.76)
Retinopathy	1.52 (0.55–4.15)	2.45 (0.96–6.27)

Odds ratios (ORs) and 95% confidence intervals (CIs) were estimated using univariate logistic regression models, with the normoglycemic group as the reference category. Outcomes were defined as the presence of at least one diabetes-related complication, peripheral neuropathy, or diabetic retinopathy, respectively. ORs > 1 indicate increased odds of the outcome compared to normoglycemic subjects. Statistical significance was set at a two-sided *p* < 0.05.

## Data Availability

The data presented in this study are available on request from the corresponding author due to privacy or ethical restrictions.

## Abbreviations

The following abbreviations are used in this manuscript:

ANOVA	Analysis of Variance
BMI	Body Mass Index
CI	Confidence Interval
HbA1c	Glycated Hemoglobin
HTN	Hypertension
OR	Odds Ratio
SD	Standard Deviation
